# H_2_O_2_ and Ca^2+^-based signaling and associated ion accumulation, antioxidant systems and secondary metabolism orchestrate the response to NaCl stress in perennial ryegrass

**DOI:** 10.1038/srep36396

**Published:** 2016-11-02

**Authors:** Tao Hu, Ke Chen, Longxing Hu, Erick Amombo, Jinmin Fu

**Affiliations:** 1 Key Laboratory of Plant Germplasm Enhancement and Specialty Agriculture, Wuhan Botanical Garden, Chinese Academy of Science, Wuhan 430074, Hubei, P.R. China; 2 Sino-Africa Joint Research Center, Chinese Academy of Science, Wuhan 430074, Hubei, P.R. China

## Abstract

Little is known about the interplay between Ca^2+^ and H_2_O_2_ signaling in stressed cool-season turfgrass. To understand better how Ca^2+^ and H_2_O_2_ signals are integrated to enhance grass acclimation to stress conditions, we analyzed the rearrangements of endogenous ion accumulation, antioxidant systems and secondary metabolism in roots, stems and leaves of perennial ryegrass (*Lolium perenne* L.) treated with exogenous Ca^2+^ and H_2_O_2_ under salinity. Ca^2+^ signaling remarkably enhanced the physiological response to salt conditions. Ca^2+^ signaling could maintain ROS homeostasis in stressed grass by increasing the responses of antioxidant genes, proteins and enzymes. H_2_O_2_ signaling could activate ROS homeostasis by inducing antioxidant genes but weakened Ca^2+^ signaling in leaves. Furthermore, the metabolic profiles revealed that sugars and sugar alcohol accounted for 49.5–88.2% of all metabolites accumulation in all treated leaves and roots. However, the accumulation of these sugars and sugar alcohols displayed opposing trends between Ca^2+^ and H_2_O_2_ application in salt-stressed plants, which suggests that these metabolites are the common regulatory factor for Ca^2+^ and H_2_O_2_ signals. These findings assist in understanding better the integrated network in Ca^2+^ and H_2_O_2_ of cool-season turfgrass’ response to salinity.

Calcium (Ca^2+^) and hydrogen peroxide (H_2_O_2_) are vital secondary messengers in plant signaling networks, and they could trigger different physiological and molecular responses to various environmental stresses in plant^1^,^2^,^3^. Ca^2+^ and H_2_O_2_ never work alone as the secondary messengers in cell signal transduction, however, most of previous studies focused on the single Ca^2+^ or H_2_O_2_ 4,5. Ca^2+^ signaling could readily regulate ion transport behavior and cell wall enzyme activities in plant cells^6^. H_2_O_2_ was mainly generated at the plant plasma membrane, and could enhance protein tyrosine phosphorylation or hyperoxidation in cytoplasm^7^. Cellular H_2_O_2_ level is controlled by a complex set of antioxidant enzymes such as catalase (CAT)^8^. Hu *et al.*^9^ reported that there was one crosstalk between Ca^2+^ and H_2_O_2_ signaling in the regulation of plant defenses. Some studies demonstrated that Ca^2+^ could activate CAT enzyme activity to minimize cellular H_2_O_2_ level, and function as an upstream component in H_2_O_2_ stress signals in plants^10^. However, Sun *et al.*^2^ suggested that H_2_O_2_ may function as an upstream signal to regulate Ca^2+^ in NaCl-stressed *Populus euphratica* cells. Despite their relevance in many cell biology aspects, integrated changes in spatio-temporal regulation of H_2_O_2_ and Ca^2+^ signaling remains elusive in stressed plants^7^,^10^,^11^.

Reactive oxygen species (ROS) accumulation, such as H_2_O_2_, HO and O_2_^−^, could be triggered by environmental stresses^12^. In plant cells, the reaction or conversion summarized by the expression: O_2_^−^ → H_2_O_2_ → HO → H_2_O is catalyzed by different antioxidant enzymes such as superoxide dismutase (SOD, EC 1.15.1.1), peroxidase (POD, EC 1.11.1.7), ascorbate peroxidase (APX, EC 1.11.1.11), catalase (CAT, EC 1.11.1.16)^13^,^14^,^15^. Generally, these enzymes have multiple molecular forms (isoenzymes) in different plant cell organelles. For example, SOD can be divided into ChlCu/ZnSOD, CytCu/ZnSOD, MnSOD, NiSOD or FeSOD isoenzymes depending on their metal cofactor, which are localized in the chloroplasts, cytosol and mitochondria. All these isoforms play cooperative role in protecting each organelle and minimizing tissue injury caused by ROS^16^,^17^. In addition, the specific isoenzymes in many plants can be recognized as the biomarkers in response to various environmental stresses, and regulated by distinct genes^12^. APX, POD and CAT are the most abundant antioxidant enzymes in detoxifying H_2_O_2_, which catalyze the reduction of H_2_O_2_ to water in plant organelles^18^. APX reduces H_2_O_2_ in chloroplasts, mitochondria, peroxisomes and apoplastic space. CAT on the other hand acts in glyoxysomes and peroxisomes, which minimize cellular H_2_O_2_ level^19^. On the contrary, high H_2_O_2_ accumulation may increase CAT activity or *CAT* gene expression^20^. Consistently, H_2_O_2_ treatment could activate Ca^2+^ channels and simultaneously mediate Ca^2+^ influx in protoplasts and increase of [Ca^2+^]cyt in intact guard cells^21^. However, Yang and Poovaiah^22^ reported that Ca^2+^/CaM could down-regulate H_2_O_2_ levels in plants by stimulating the catalytic activity of plant CAT. Jiang and Huang^23^ showed that Ca^2+^ could increase CAT activity in heat stressed grasses. The regulatory mechanisms among Ca^2+^ and H_2_O_2_ signaling, and antioxidant enzymes also remain unclear, while several lines of evidence indicate one interaction between their multiple signal transduction.

Many studies have revealed that regulation of Ca^2+^ and H_2_O_2_ signaling involves many genes and metabolites in stressed plant^5^,^22^,^24^. Using gas chromatography-mass spectrometry (GC-MS) analysis of polar compounds, Shi *et al.*^5^ found that 42 metabolites including amino acids, organic acids, sugars, and sugar alcohols were regulated by exogenous Ca^2+^ treatment in bermudagrass [*Cynodon dactylon* (L.) Pers.] in response to cold stress. Dittami *et al.*^24^ showed that the accumulation level of mannitol, n-3 to n-6 ratio of polyunsaturated fatty acids and 22 amino acids changed after H_2_O_2_ treatment in brown alga *Ectocarpus siliculosus*. Furthermore, previous studies have demonstrated that the majority of the expressed genes could be activated by these metabolites, which may be the gene regulation end products^25^. Furuichi *et al.*^26^ reported that the increase of [Ca^2+^]_cyt_ might initiate the expression of genes, such as *AtSUC1* and *AtSUC2*, involved in biosynthesis of storage carbohydrate. On the contrary, exogenous application of hexoses also might mediate by monosaccharide-H^+^ co-transporters to increase [Ca^2+^]_cyt_ level. In addition, H_2_O_2_ signaling activating gene expression also involved metabolites such as proline and sucrose^8^. Therefore, we speculated that the changes in metabolites abundance involved in the crosstalk between Ca^2+^ and H_2_O_2_ signaling may be regarded as a major regulation character in plants in response to stress environment.

Salinity is a major abiotic stress and increasingly threatens agriculture and food production worldwide^27^. Perennial ryegrass (*Lolium perenne* L.) is an extensively utilized cool-season grass globally used for forage, turfgrass and soil stabilization in United States, Australia, New Zealand, Japan and most of European countries because of its rapid establishment rate^28^. Our previous study confirmed that salt stress could cause oxidative injury and damage to cell membrane integrity in perennial ryegrass, and antioxidant enzymes decreased the stress toxicity^12^,^17^. In the present study, we further investigated the impact of exogenous Ca^2+^ or H_2_O_2_ on the secondary metabolism and oxidative signaling in perennial ryegrass under salt stress. The objective of this study was to further understand the orchestrated response of antioxidant system and metabolisms induced by H_2_O_2_/Ca^2+^ signaling in cool-season turfgrass in response to salt stress.

## Results

### The impact of exogenous Ca^2+^ on physiological features of salt-stressed perennial ryegrass

**Figure 1:**
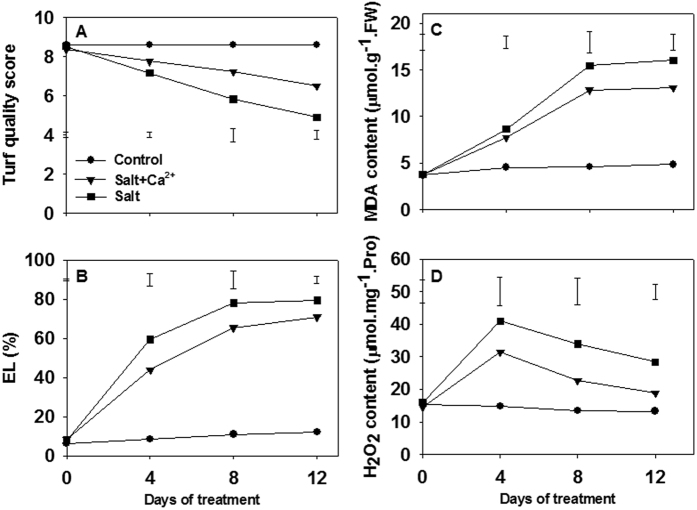
Effect of salt stress on physiological responses in perennial ryegrass. (**A**) turf qualiy; (**B**) electrolyte leakage (EL); (**C**) MDA content; (**C**) H_2_O_2_ content. Vertical bars at the top indicate LSD values (*P* < 0.05) for the comparison of different treatments on a given day of treatment.

As shown in Fig. 1, 300 mM NaCl treatment enhanced the levels of MDA, EL and H_2_O_2_, but minimized the turf quality score in perennial ryegrass leaves. However, 7 mM Ca(NO_3_)_2_·4H_2_O added in 300 mM NaCl treatment solution reduced the levels of MDA, EL and H_2_O_2,_ and increased the turf quality score compared with 300 mM NaCl treated plants (Fig. 1). These alleviated injury features in salt-treated plants induced by Ca^2+^ could be observed from the fourth and eighth day treatment (Supplementary Figure S1).

### The impact of exogenous Ca^2+^ on endogenous ion accumulation in salt-stressed perennial ryegrass

**Figure 2:**
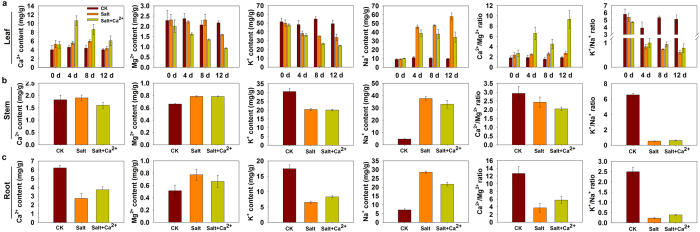
External application of Ca^2+^ induces the changes of sodium (Na), potassium (K), calcium (Ca), magnesium (Mg), Ca^2+^/Mg2^+^ and K^+^/Na^+^ ratio in salt-stressed perennial ryegrass. Vertical bars represent means ± standard errors (n = 4) based on least significant difference (LSD) test (*P* < 0.05).

Treatment with Ca^2+^ under salinity enhanced higher Ca^2+^ content in the leaves than that treated with NaCl alone (Fig. 2a). In the roots, NaCl treatment decreased the Ca^2+^ content compared with the controls, but exogenous application of Ca^2+^ relieved the decrease (Fig. 2c). In the leaves, external application of Ca^2+^ declined Mg^2+^ content at 4, 8 and 12 DAT, but treatment with NaCl alone stimulated the content decline at 12 DAT (Fig. 2a). In the stem, Mg^2+^ content increased under both salt treatment and salt^+^ Ca^2+^ treatment (Fig. 2b). The increased Mg^2+^ content in root was just found in treatment line with NaCl alone (Fig. 2c). For K^+^, NaCl treatment decreased the content in leaf, stem and root, but exogenous application of Ca^2+^ relieved the decrease in leaf. In the NaCl treated leaf, stem and root of perennial ryegrass, endogenous Na^+^ content showed remarkable increase but the increase was inhibited under the exogenous application of Ca^2+^. In addition, the NaCl treatment enhanced Ca^2+^/Mg^2+^ ratio in leaf but reduced it in stem and root. Exogenous application of Ca^2+^ under salinity caused a higher Ca^2+^/Mg^2+^ ratio in leaf and root compared with NaCl treatment alone. The K^+^/Na^+^ ratio decreased in leaf, stem and root in response to NaCl treatment. Exogenous application of Ca^2+^ relieved the decrease in leaf at 8 DAT and in NaCl-stressed root.

### The impact of exogenous Ca^2+^ on the antioxidant systems of salt-stressed perennial ryegrass

**Figure 3:**
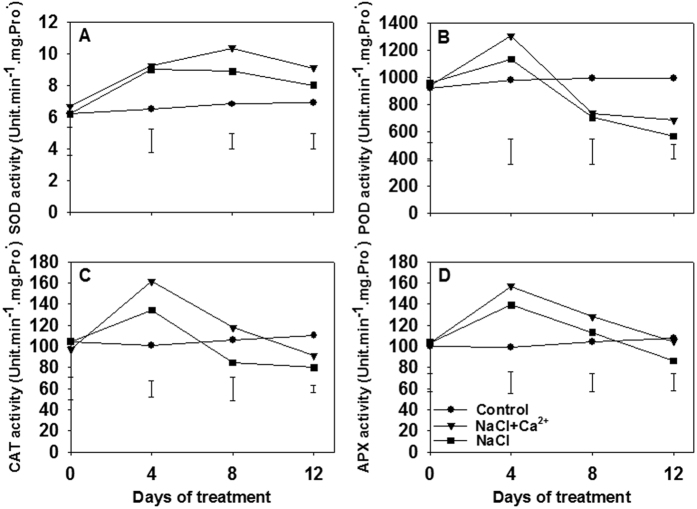
Exogenous Ca^2+^ modulates antioxidant-related enzymatic activities in perennial ryegrass. Effect of exogenous Ca^2+^ in the activity of SOD (**A**), POD (**B**), CAT (**C**) and APX (**D**). The plants were treated with 300 mM NaCl + 7 mM Ca(NO_3_)_2_·4H_2_O (T1) or 300 mM NaCl (T2) at 0, 4, 8 and 12 d. Vertical bars at the top indicate LSD values (*P* < 0.05) for the comparison of different treatments on a given day of treatment.

To investigate the salinity response induced by Ca^2+^ signal, the SOD, POD, CAT and APX activities, as well as oxidative stress-responsive isoenzymes and genes were analyzed (Fig. 3). During the 12 days treatment, salinity stress induced greater SOD activity in leaves compared with controls, however, Ca^2+^ addition to NaCl-treated plants induced more pronounced increase at 8 and 12 DAT (Fig. 3a). An increase in POD and CAT activities was observed at 4 DAT, while the activities were depressed at 8 and 12 DAT. Application of Ca^2+^ upon salinity imposition enhanced POD activity at 4 and 12 DAT, and CAT activity at 4, 8 and 12 DAT respectively in comparison with NaCl only treatment (Fig. 3b). Salinity increased leaf APX activity at 4 DAT but decreased the activity at 12 DAT. Exogenous application of Ca^2+^ in salt-treated leaves induced higher APX activity compared with NaCl treatment alone at 4, 8 and 12 DAT. The changes of antioxidant isoenzymes for SOD, POD, CAT and APX confirmed these discovery on enzyme activity (Supplementary Figure S2).

### The impact of H_2_O_2_ and Ca^2+^ signaling on the oxidative status in salt-stressed perennial ryegrass

**Figure 4:**
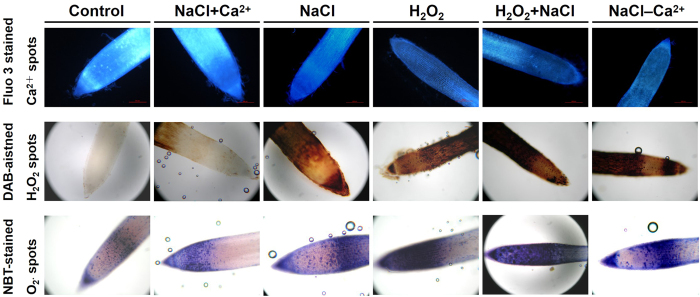
Histochemical analysis for the generation of Ca^2+^, H_2_O_2_ and O_2_^−^ in perennial ryegrass roots.

To elucidate further the integrated connection between exogenous H_2_O_2_/Ca^2+^ and oxidative protection against salinity stress, endogenous H_2_O_2_ and Ca^2+^ steady state levels were monitored in perennial ryegrass roots at 8 DAT (Fig. 4). Treatment with NaCl alone decreased Ca^2+^ accumulation, but stimulated the H_2_O_2_ and O_2_^−^ production. H_2_O_2_ and O_2_^−^ content also increased remarkably, and Ca^2+^ accumulation declined in H_2_O_2_ alone treated plants. In addition, treatment with both NaCl and H_2_O_2_ induced higher H_2_O_2_ and O_2_^−^ accumulation and lower Ca^2+^ content. However, in the presence of NaCl, greater Ca^2+^ accumulation and lower H_2_O_2_ and O_2_^−^ production were observed because of exogenous Ca^2+^ application. In contrast, depriving Ca^2+^ from the 300 mM NaCl treatment solution induced higher H_2_O_2_ and O_2_^−^ accumulation and lower Ca^2+^ production compared with NaCl treatment alone (Fig. 4).

**Figure 5:**
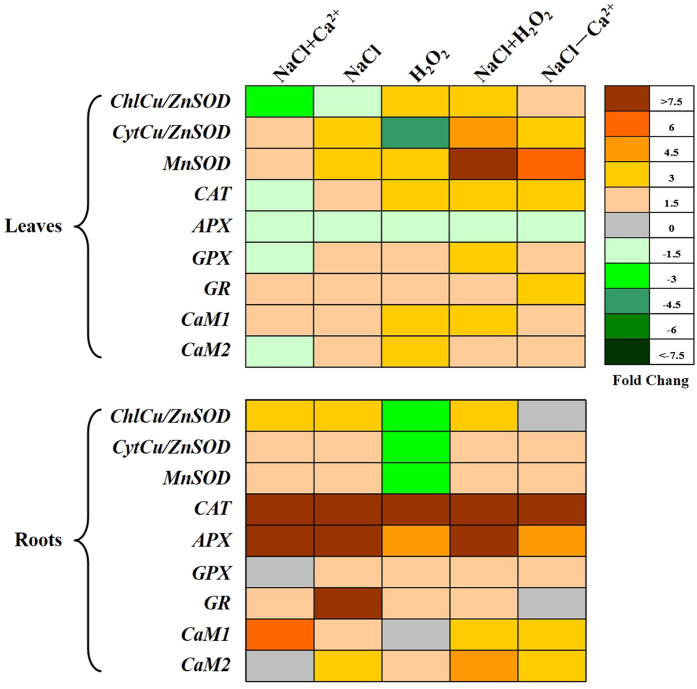
H_2_O_2_- and Ca^2+^ -associated gene expression is regulated in a tissue and time-specific manner in perennial ryegrass. Heat diagrams showing the temporal expression pattern in selected genes associated with H_2_O_2_ and Ca^2+^ production in leaves and roots of perennial ryegrass under salt stress.

Meanwhile, to further assess the role of H_2_O_2_ and Ca^2+^ signaling in the ROS metabolism, expression profiles of calmodulin binding protein genes (*CaM1* and *CaM2*) and ROS metabolism genes (*ChlCu/ZnSOD*, *CytCu/ZnSOD*, *MnSOD*, *CAT*, *APX*, *GPX* and *GR*) were analyzed in perennial ryegrass leaves and roots. When exposed to all treatments, the transcripts levels of *APX* remained down-regulated in leaves (Fig. 5). Under NaCl treatment, *CytCu/ZnSOD*, *MnSOD*, *CAT*, *GPX* and *GR* increased transcript abundant but *ChlCu/ZnSOD* was down-regulated in the leaves. All ROS genes were up-regulated in the salt-stressed roots Upon Ca^2+^ addition, *ChlCu/ZnSOD*, *CAT*, *APX* and *GPX* were down-regulated compared with control. However, application of H_2_O_2_ or depriving Ca^2+^ from NaCl treatment induced all ROS genes expression in leaves, except for *CytCu/ZnSOD* and *APX*. In roots, most of ROS genes also showed increased transcripts levels compared with control. Upon Ca^2+^ addition, *CaM1* was induced but *CaM2* inhibited in both leaves and roots. However, *CaM2* showed higher transcripts than *CaM1* in roots after application of H_2_O_2._ For other treatment lines, CaM1 and *CaM2* were induced in both leaves and roots.

### The impact of H_2_O_2_ and Ca^2+^ signaling on the metabolite profiles in salt-stressed perennial ryegrass

**Figure 6:**
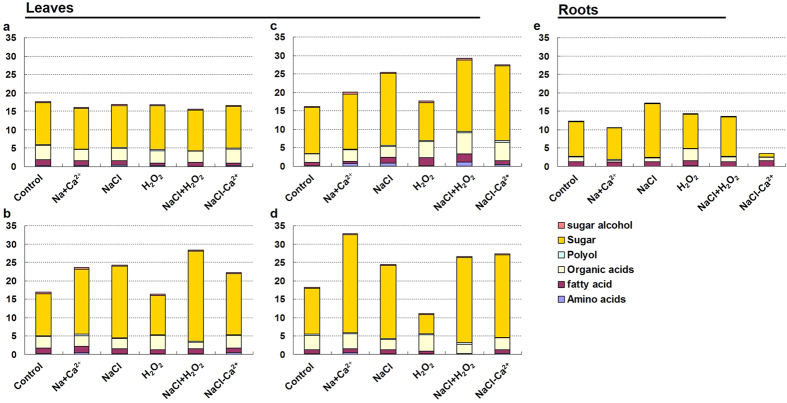
Evaluation of metabolite targets accumulation signatures in perennial ryegrass plants. (**a–d**) represent the stressed samples for 0, 2, 4 or 8 days, respectively. Experiments were repeated at least three times, each with three replicates, and *P*-value was calculated by LSD’ t-test.

To identify candidate metabolites targets in salt-stressed plants, the H_2_O_2_ and Ca^2+^ treated samples were extracted and derivatized, and then analyzed by GC-MS. A total of 160 total peaks with fairly consistent retention times (RT) and excellent resolution were resolved from each polar extract. Based on the internal consistency of RT and retention indices (RI), 41 metabolites could be identified across all samples in this study (Supplementary Table S2). The identified metabolites included 15 organic acids, 12 sugars, 8 amino acids, 4 fatty acids and 2 polyols (Fig. 6; Supplementary Table S3). Each metabolite in each treatment was given in Table S3.

Under NaCl treatment, the total content of 41 metabolites increased in perennial ryegrass leaves. Upon Ca^2+^ addition, more metabolites accumulated at 8 DAT (Fig. 6d). However, H_2_O_2_ stress decreased the 41 metabolites total content after 2 days treatment (Fig. 6c,d). Both NaCl and H_2_O_2_ chemical treatments stimulated greater metabolites accumulation compared with NaCl or H_2_O_2_ application alone (Fig. 6c,d). Removing Ca^2+^ from NaCl treatment induced lower accumulation at 2 DAT (Fig. 6c) but higher metabolites content at 4 and 8 DAT (Fig. 6b–d) compared with NaCl treatment in leaves. However, treatments without Ca^2+^ in NaCl solution decreased the metabolites content in roots (Fig. 6e). In addition, treatments with NaCl or H_2_O_2_ increased the content of metobolites, but upon Ca^2+^ addition, metabolite accumulation declined in roots (Fig. 6e).

### The impact of H_2_O_2_ and Ca^2+^ signaling on sugars and sugar alcohol content in salt-stressed perennial ryegrass

**Figure 7:**
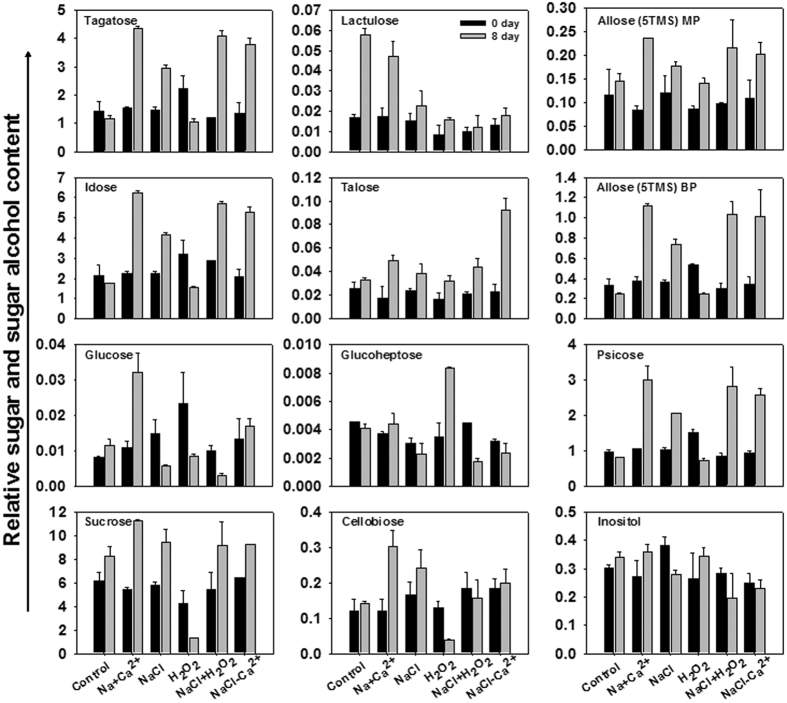
H_2_O_2_ and Ca^2+^ signaling associated content of sugars and sugar alcohol in plant leaves aftert 8 days streatment. Vertical bars indicate standard error of each mean for 11 sugars and one sugar alcohol (Inositol) at a given day of treatment. Experiments were repeated at least three times, each one with three replicates, and *P*-value was calculated by LSD’ t-test.

The increase of fatty acid, sugars and organic acids content was the major contributor of total metabolites content increase in leaves (Fig. 6). Eleven sugars and one sugar alcohol accounted for more than 66.0% of total metabolites content except H_2_O_2_ chemical treatments only 49.5% at 8 DAT. By further evaluating the effects of H_2_O_2_ and Ca^2+^ signaling on sugars and sugar alcohol content in detail, we found that all the twelve metabolites were highly accumulated after Ca^2+^ application compared with control and NaCl treatment lines (Fig. 7). With NaCl treatment, tagatose, idose, sucrose, talose, allose (5TMS) BP and psicose showed higher content compared with controls. However, NaCl treatment induced lower glucose and inositol content compared with controls. Tagatose, idose, glucose, talose and psicose content had higher content by depriving Ca^2+^ from NaCl treatment than by NaCl treatment alone in leaves (Fig. 7). H_2_O_2_ treatment induced lower accumulation of tagatose, idose, glucose, sucrose, cellobiose, psicose and allose (5TMS) BP, but greater content of talose, glucoheptose and allose (5TMS) MP in comparison with controls. In contrast, both NaCl and H_2_O_2_ chemical treatments stimulated higher abundance of tagatose, idose, sucrose, psicose and allose (5TMS) BP, but lower glucoheptose abundance compared with controls (Fig. 7).

**Figure 8:**
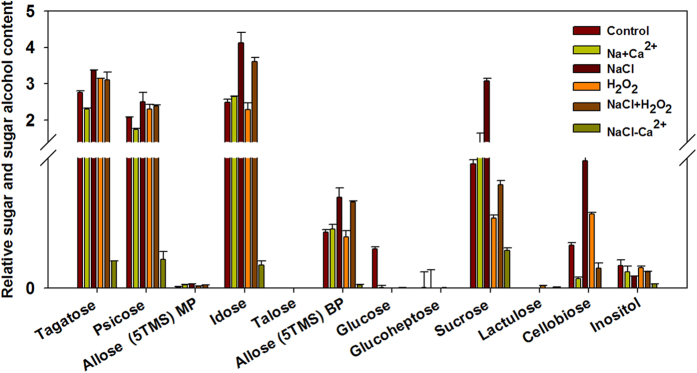
H_2_O_2_ and Ca^2+^ signaling associated content of sugars and sugar alcohol in plant roots aftert 8 days streatment. Vertical bars indicate standard error of each mean for 11 sugars and one sugar alcohol (Inositol) at a given day of treatment. Experiments were repeated at least three times, each one with three replicates, and *P*-value was calculated by LSD’ t-test.

In the roots, we found that glucose abundance decreased significantly in all treatment lines (Fig. 8). NaCl stress increased tagatose, psicose, idose, allose (5TMS) BP, sucrose and cellobiose content. Interestingly, adding and removing Ca^2+^ from NaCl solution induced similar effect. Tagatose, psicose, idose, allose (5TMS) BP, sucrose, cellobiose and inositol abundance had lower accumulation compared with NaCl stress alone, but eventually declined. In addition, plant roots accumulated lower tagatose, psicose, idose, allose (5TMS) BP, glucose, sucrose, cellobiose and inositol under NaCl treatment without Ca^2+^ compared with controls. However, the treatment after Ca^2+^ addition increased sucrose content (Fig. 8). H_2_O_2_ treatments decreased glucose and sucrose, but increased cellobiose abundance. Both NaCl and H_2_O_2_ chemical treatments stimulated higher idose and allose (5TMS) BP abundance, but lower glucose, sucrose and cellobiose accumulation. Idose, allose (5TMS) BP, sucrose showed greater abundance under both NaCl and H_2_O_2_ chemical treatments than H_2_O_2_ alone. In contrast, both NaCl and H_2_O_2_ chemical treatments induced lower cellobiose content than H_2_O_2_ alone (Fig. 8).

## Discussion

In this study, we initially characterized the impact of external Ca^2+^ or H_2_O_2_ on the physiological status of salt-treated cool-season turfgrass. Exogenous administration with Ca^2+^ alleviated the physiological damage induced by salt tress, as shown by the higher turf quality and lower EL, MDA and H_2_O_2_ content. This confirmed previous observations that Ca^2+^ treatment significantly improved the physiological response of stressed plants^5^. Similar results in the Ca^2+^ induced physiological behavior in stressed plants were previously found in tall fescue^23^, which suggest that Ca^2+^ acts on key signaling in the stress response pathways of cool-season turfgrass^29^. Furthermore, we found that the application of external H_2_O_2_ could trigger Ca^2+^ signals positively involved in the rearrangements of ROS accumulation and metabolite profiles.

To elucidate the mechanism of salt tolerance induced by external Ca^2+^ application and transport, we monitored internal levels of Ca^2+^, Mg^2+^, K^+^ and Na^+^ in NaCl stressed root, stem and leaf. We found that Ca^2+^ pool in leaves and roots is modulated in response to NaCl stress. NaCl treatment decreased Ca^2+^ accumulation in root, but exogenous application of Ca^2+^ relieved the decline. In the leaves, no significant difference was obtained between controls and NaCl treated line, however, a higher Ca^2+^ content was found after treatment with Ca^2+^ under salinity. These results suggest that Ca^2+^ metabolism is regulated by salinity, which was similar with heat^23^,^30^ and cold stresses^5^. In addition, the Ca^2+^ pool influx into leaves under salt stress may be a crucial salinity acclimation mechanism for perennial ryegrass. Our previous study had confirmed that the changes of macronutrient cations content, such as Ca^2+^, Mg^2+^, Na^+^, and K^+^, are necessary for salinity adaptation of perennial ryegrass^12^. In this study, NaCl treatment decreased K^+^ but increased Na^+^ concentration in leaves, stems and roots, which is consistent with our previous studies^12^,^31^. In roots, external Ca^2+^ application significantly increased K^+^ concentration but decreased Na^+^ concentration. Even though Ca^2+^ treatment did not increase K^+^ concentration in leaves, Na^+^ concentration was still lower compared with NaCl treatment alone. Moreover, external Ca^2+^ application significantly induced higher K^+^/Na^+^ ratio in roots at 8 days treatment leaves. Evidence has shown that regulation of K^+^ uptake and prevention of Na^+^ influx to maintain desirable K^+^/Na^+^ ratios in the cytosol are vital strategies of improving plant salt tolerance^32^. These results suggest that increasing the K^+^/Na^+^ ratio could be one regulation mechanism induced by Ca^2+^ signaling in salt stressed perennial ryegrass. Meanwhile, Mg^2+^ concentration decreased at 12 DAT in leaves, but increased in stems and roots. Interestingly, feeding salt-treated leaves with Ca^2+^ induced lower accumulation of Mg^2+^ and Na^+^ compared with the NaCl treatment alone. A higher Ca^2+^/Mg^2+^ ratio were observed in leaf and root than NaCl-stressed tissues. Low Ca^2+^/Mg^2+^ quotient in culture media is often thought as a restriction factor to restrict species from growing in these culture conditions^33^. We could deduce that the increase of Ca^2+^/Mg^2+^ ratio could be one adaption mechanism induced by Ca^2+^ signaling in salt stressed perennial ryegrass.

Some protective antioxidant enzymes/genes and metabolites are the backbone of the oxidative status in plants^34^,^35^. Although salinity conditions produced excessive H_2_O_2_ and O_2_^−^ in plants, the higher antioxidant enzymes activity including SOD, POD, CAT and APX inhibited the excessive ROS accumulation to maintain ROS homeostasis^12^,^36^, and our enzymes activity data support this. Ca^2+^ signals perform functions upstream or downstream in the ROS signal transduction network in plants^10^. Notably, Ca^2+^ application under salinity inhibited the decrease of enzyme activity, and greater SOD, POD, CAT and APX activity could be obtained when exogenous application of Ca^2+^ in NaCl solution. These data are in agreement with that Ca^2+^ signaling could improve the stress adaptation by stimulating higher antioxidant enzymes activity in plants^23^, which were further confirmed by greater isoenzymes intensity of SOD (3–5), POD (2–5), CAT (1–2), APX (1–5) (Supplementary Figure S2). The increased antioxidant enzymes activity might be attributed to up/down regulated expression of the candidate genes^17^,^34^. Consistently, this study also observed that the expression of *ChlCu/ZnSOD*, *CytCu/ZnSOD*, *MnSOD*, *CAT*, *APX*, *GPX* and *GR* changed under salt stress. These were involved in the regulation of antioxidant enzyme activities. NaCl direct treatment increased transcript abundance of *CytCu/ZnSOD*, *MnSOD*, *CAT*, *GPX* and *GR* in leaves. In contrast, Ca^2+^ addition treatment induced the up-regulation of only three genes *CytCu/ZnSOD*, *MnSOD* and *GR* in the leaves. However, in roots, *ChlCu/ZnSOD*, *CytCu/ZnSOD*, *MnSOD*, *CAT*, *APX* and *GR* were up-regulated under exogenous Ca^2+^ application in NaCl treatment, indicating that the positive role in the Ca^2+^ dependent regulation of antioxidant enzyme gene was greater in root than in leaf under salt stress. These results were confirmed by the opposite changes detected in roots and leaves after removing Ca^2+^ from NaCl treatment.

Both Na^+^ and Ca^2+^ were added into half-strength Hoagland’s solution and first affected the normal functioning of the root in our experiment. H_2_O_2_ forms the basis of the ROS signals mediated priming phenomenon in stressed plants^37^. To understand further the mechanistic connection between H_2_O_2_ and Ca^2+^ signaling in response to salt stress, we investigated the endogenous steady state of Ca^2+^, H_2_O_2_ and O_2_^−^ levels in roots. Results obtained further proved that exogenous application of Ca^2+^ in NaCl treatment had higher intensity of Ca^2+^ signaling in plant tissues than NaCl treatment alone^38^. Compared with direct NaCl stress, the weaker DAB and NBT staining for H_2_O_2_ and O_2_^−^ after exogenous application of Ca^2+^ under stress conditions was observed. Moreover, removing all Ca^2+^ from the NaCl treatment solution induced higher H_2_O_2_ and O_2_^−^ accumulation and lower Ca^2+^ production compared with NaCl treatment alone, indicating exogenous Ca^2+^ modulates salinity-induced oxidative damage by directly scavenging H_2_O_2_ and O_2_^−^. Consistently, H_2_O_2_ could function as an upstream or downstream component in the Ca^2+^ signaling network^2^. The paper reported that H_2_O_2_ induced [Ca^2+^]_cyt_ increased in leaf cells^39^. However, in our study, H_2_O_2_ treatment alone decreased Ca^2+^ signaling and increased DAB and NBT staining for H_2_O_2_ and O_2_^−^ in roots. In addition, treatment with both NaCl and H_2_O_2_ induced lower Ca^2+^ content but higher accumulation of H_2_O_2_ and O_2_^−^, indicating H_2_O_2_ in roots may be one negative regulator of Ca^2+^ signaling pathway in response to salinity stress. Calmodulin (CaM) genes, such as *CaM1* and *CaM2*, could regulate different target intermediate in Ca^2+^ mediated signal transduction^40^. Data from the current experiments showed that H_2_O_2_ stress induced *CaM1* and *CaM2* expression in leaves. However, the H_2_O_2_ stressed roots exhibited the same *CaM1* transcript level as the controls. Consistently, the strong induction of *CaM1* and *CaM2* in NaCl stressed leaves and roots reinforces the recent suggestion that H_2_O_2_ may function as an upstream signal activating Ca^2+^ signals in NaCl stressed perennial ryegrass^2^.

H_2_O_2_ is one of the major active oxygen species and could be synthesized by the catalyzed reaction of SOD^34^. Our results demonstrated that salt stress increased the H_2_O_2_ level while SOD activity and *CytCu/ZnSOD* and *MnSOD* expression level increased in leaves of perennial ryegrass. In salt-stressed roots, the H_2_O_2_ level significantly increased with an increasing expression of *ChlCu/ZnSOD*, *CytCu/ZnSOD* and *MnSOD*. Excessive H_2_O_2_ could be detoxified into H_2_O by the catalyzed reaction of CAT, GPX, APX or GR in plants cells^41^. Our previous study showed that NaCl stress increased the transcript abundance of *CAT*, *GPX*, *APX* or *GR*^12^. Achieving ROS homeostasis is necessary self-defense mechanism for plant in response to stress conditions^42^. Here, qPCR analysis showed that *CAT* and *APX* had higher transcript abundance than *SOD* genes such as *ChlCu/ZnSOD*, *CytCu/ZnSOD* and *MnSOD* in NaCl-stressed root. The same result was obtained under H_2_O_2_ treatment and H_2_O_2_^+^ NaCl treatment, suggesting that self-regulation for ROS homeostasis could be activated due to the excessive accumulation of H_2_O_2_ induced by NaCl stress. Together with exogenous Ca^2+^ signal could increase SOD, POD, CAT and APX activity, as well as may function as a downstream signal activated by H_2_O_2_, we speculated that Ca^2+^ and H_2_O_2_ signals may exist one common regulation orientation that maintains ROS homeostasis in salt-stressed cool-season turfgrass.

Since the accumulation of various metabolites changed when plants were exposed to salinity conditions^43^, metabolite profile analysis was performed in leaf and root samples to characterize how Ca^2+^ and H_2_O_2_ signals affect metabolite patterns in salt-stressed cool-season turfgrass. Here, 41 metabolites exhibited different response patterns in plants exposed to NaCl, Ca^2+^ or H_2_O_2_. Consistent with the previous research in barley (*Hordeum vulgare* L.)^43^ and *Arabidopsis thaliana*^44^, the accumulation of amino acids, organic acids and sugars changed in salt-stressed perennial ryegrass. Metabolites may therefore play vital roles in salinity response pathway. In addition, the present study also characterized a number of modified metabolites that were not observed in previous salinity metabolic response studies, such as polyol and fatty acids. Compared with NaCl treatment alone, the total content of 41 metabolites showed upward trend upon Ca^2+^ addition during the first 4 days exposure. In contrast, removing Ca^2+^ or adding H_2_O_2_ accelerated the upward trend. At 8 DAT, upon Ca^2+^ addition treatment obtained the highest accumulation compared with other treatment, but H_2_O_2_ alone treatment had the lowest metabolic accumulation during the 8 days treatment. The present data combined with previous findings involved in metabolites response under salt stress, may help to understand better the molecular basis of Ca^2+^ and ROS signaling in salt-treated plants.

Sugars not only directly provide energy and solutes for osmotic adjustment^45^,^46^, but also act as sugar-sensing signal to regulate key candidate gene expression^47^. Our metabolomic data demonstrated that the sugar and sugar alcohol contents accounted for 49.5–88.2% of all 41 metabolites accumulation in treated leaves, which lead us to hypothesize that sugars profiles is involved in sensing Ca^2+^ and ROS signaling in salt-treated leaves. First, NaCl treatment induced the production of tagatose, idose, sucrose, talose, allose (5TMS) BP and psicose but inhibited glucose and inositol content in leaves. However, we found that all eleven sugars and one sugar alcohol showed higher accumulation after Ca^2+^ application compared with control or NaCl treatment lines in leaves. On the contrary, NaCl treatment induced higher sugar than that in Ca^2+^ application lines in root. These results suggest that up-regulated sugars induced by Ca^2+^ signaling in leaf could contribute to the stronger response to salt stress. Secondly, the lowest contribution rate (49.5%) for sugars in total metabolites content was just obtained at H_2_O_2_ treatment alone at 8 DAT. Correspondingly, in leaves, we found that H_2_O_2_ treatment alone induced lower accumulation of tagatose, idose, glucose, sucrose, cellobiose, psicose and allose (5TMS) BP compared with controls or NaCl treatment lines, and just the talose, glucoheptose and allose (5TMS) MP content increased, which indicated that the positive role induced by Ca^2+^ signaling in leaf may be inhibited by H_2_O_2_ signaling. However, converse results were observed in stressed roots, which may be in relation to the different carbohydrate allocation in source and sink tissue under salt stress^46^.

In conclusion, the present study reported here reinforces the understanding of salt tolerance involved in H_2_O_2_ and Ca^2+^-based signaling response through coupling ion accumulation, antioxidant systems and secondary metabolism analysis in cool-season turfgrass. Increased Ca^2+^/Mg^2+^ ratio but decreased Na^+^/K^+^ ratio were observed following external Ca^2+^ application in salt-stressed grass. Exogenous Ca^2+^ signaling could induce higher antioxidant enzymes activity and the expression level of antioxidant gene and protein in plants to adapt salinity environment. However, H_2_O_2_ application decreased Ca^2+^ signaling but increased the transcript abundance of *CAT* and *APX*. In order to maintain ROS homeostasis, Ca^2+^ and H_2_O_2_ signals had one common regulation pattern in salt-stressed cool-season turfgrass. In addition, H_2_O_2_/Ca^2+^-mediated metabolites detected in this study could provide a dataset of common regulatory factors for signaling transduction and salinity acclimation in perennial ryegrass. These findings involved in overlapping roles of H_2_O_2_/Ca^2+^ signaling in salinity response mechanisms supply one novel strategy for a cool-season turfgrass’s adaptation under salt stress.

## Methods

### Plant materials and growth conditions

Perennial ryegrass ‘Quick start II’ seeds were planted into plastic pots (10 cm diameter, 15 cm deep) with sand as substrate. After seedlings had grown to 8 cm tall, they were mowed to a height of 5 cm and were irrigated twice weekly with half-strength Hoagland’s solution^48^. Forty-day-old seedlings were rinsed thoroughly using distilled water, and transferred into 300 mL Erlenmeyer flasks filled with approximately 290 mL half-strength Hoagland’s solution. The bottlenecks were closed with appropriate amount of absorbent paper twined using plastic food wrap. To prevent potential algal growth, the flasks were wrapped with aluminum foil. The nutrient solutions were completely replaced each week. All plants were grown under greenhouse conditions (daily temperature of 2471/20711C (day/night), photosynthetic active radiation (PAR) at 300 μmol m^−2^s^−1^, and a 14 h photoperiod) throughout the experiment.

## Treatments

### Experiment 1

Four weeks after canopy and root system establishment, plants were separated into three groups (Group I, II, III) according to the similar transpiration rate calculated based on the weight difference at three-day intervals before salt treatment initiation described by Hu *et al.*^12^. Group I plants were supplied with half-strength Hoagland’s solution throughout the whole experimental procedure as control line. Group II plants, as salt treatment line, were treated with 300 mM NaCl for 12 d. Group III plants, as Ca^2+^ treatment line, were treated with 300 mM NaCl + 7 mM Ca(NO_3_)_2_·4H_2_O for 12 d. All treatment solutions were prepared in half-strength Hoagland’s solution. Shoots samples for physiological, gene expression and metabolic analysis were harvested at 0, 4 d, 8 d and 12 d after treatment (DAT), respectively. Roots samples were harvested at the end of experiment. Treatments were arranged as a completely randomized design and each treatment having four plant-pot systems as four replicates.

### Experiment 2

The growing conditions were similar with those of Experiment 1. Plants were separated into six groups with the same method as shown in Experiment 1. The six treatments arranged in a completely randomized block design with four replicates were as follows: (1) nutrient solution (control); (2) 300 mM NaCl + 7 mM Ca(NO_3_)_2_·4H_2_O; (3) 300 mM NaCl; (4) H_2_O_2_; (5) H_2_O_2_ + 300 mM NaCl; (6) 300 mM NaCl- Ca^2+^ (prepared in half-strength Hoagland’s nutrient solution without Ca ion). All reagents were dissolved in half-strength Hoagland’s nutrient solution. Shoot samples were harvested at 0, 12 h, 2 d, 4 d, 8 d and 12 d after treatment, respectively. Roots samples were harvested at the end of experiment.

## Measurements

### Cell membrane stability and turf quality

Cell membrane stability was determined by the electrolyte leakage (EL) level measured based on the method described by Hu *et al.*^17^. Fresh 0.1 g of uniform leaf segments were washed with deionized water, incubated in 15 mL deionized water, and then shaken for 24 h at room temperature. The initial conductance (*C*_*i*_) was measured using a conductance meter (JENCO-3173, Jenco Instruments, Inc., San Diego, CA, USA). The samples were then autoclaved at 120 °C for 20 min to completely disrupt the tissues and to release all electrolytes. After cooling to room temperature, the conductance of the incubation solution with killed tissues (*C*_max_) was determined. Relative EL was calculated using the formula: EL (%) = (*C*_i_/*C*_max_) × 100.

Turf quality was rated visually based on turfgrass color (percentage green leaves), plant density and degree of leaf wilting on a scale of 0 to 9, where 0 score indicated withered, yellow, thin or dead grass, while 6 indicated minimum acceptable level. A 9 score indicated green, dense and uniform grass^49^.

### Antioxidant enzymes activities, cell lipid peroxidation and H_2_O_2_ level

Extract preparation and assays for SOD (EC 1.15.1.1), CAT (EC 1.11.1.6), POD (EC. 1.11.1.7), APX (EC 1.11.1.11), soluble protein, MDA and H_2_O_2_ level were conducted according to methods reported previously^17^. The SOD activity was measured according to the method of Jiang and Huang^23^. A 3 mL reaction mixture, composed of 50 mM phosphate buffer solution (PBS) (pH 7.8), 60 mM Riboflavin, 195 mM Met, 3mmM EDTA, 1.125 mM NBT and 0.1 mL enzyme extract, was placed under light at 3000 lux for 10 min, and was recorded at 560 nm absorbance by spectrophotometer (UV-2600, UNICO Instruments Co., Ltd., Shanghai, China). One unit of SOD activity was defined as the amount of enzyme that inhibited 50% photochemical reduction of NBT. POD activity was measured as an increase in absorbance at 470 nm for 1 min following the oxidation of guaiacol^50^. The reaction mixture contained 0.1 M HAC–NaAC buffer (pH 5.0), 20 mM guaiacol, 10 mM PBS (pH 7.0), 40 mM H_2_O_2_ and 0.1 mL enzyme extract. CAT activity was determined according to Chance and Maehly^51^. A reaction mixture of 50 mM PBS (pH 7.4), 45 mM H_2_O_2_ and 0.1 mL enzyme extract was measured at 240 nm absorbance for 1 min. CAT activity was determined through the decomposition of H_2_O_2_. APX activity was detected using the method described by Mittler and Zilinskas^52^.

Cell lipid peroxidation was determined by the MDA content measured as follows. One milliliter of enzyme extract was mixed with 2 ml of reaction solution containing 20% (v/v) trichloroacetic acid and 0.5% (v/v) TBA. The mixture was heated in a water bath at 95 °C for 30 min, then cooled quickly in ice-water bath to room temperature, and centrifuged at 14,000 rpm for 20 min. Absorbance of the supernatant was measured at 532 and 600 nm. MDA content was calculated based on subtracting the absorption at 600 nm from the absorption at 532 nm and calibrated with the extinction coefficient of 155 mM^−1^cm^−1^.

H_2_O_2_ level was determined based on the method described by Lin and Kao^53^. One milliliter of supernatant was mixed thoroughly with 1 ml of 0.1% titanium sulphate in 20% H_2_SO_4_ (v/v), and then centrifuged at 6000 × g for 15 min at room temperature. The supernatant was measured at 410 nm absorbance. The H_2_O_2_ level was calculated according the standard curve generated with known concentrations of H_2_O_2_ and calibrated by extinction coefficient of 0.28 μmol^−1^cm^−1^.

### RNA isolation, cDNA synthesis and real-time PCR analysis

Total RNA extraction was performed from the leaves and roots using Trizol reagent (Invitrogen, Paisley, UK) according to the manufacturer’s instructions and then was incubated with RNase-free DNase (RQ1; Promega, Madison, WI, USA). RNA integrity was examined at 260 and 280 nm by spectrophotometer (UV-2600; UNICO Instruments, Shanghai, China) and checked on a gel electrophoresis in 1.5% agarose gels with 1 μL RNA (=0.5 μg μL^−1^). Subsequently, cDNA synthesis was performed from 2 μg purified RNA using cDNA synthesis kit according to the manufacturer’s protocol (Fermentas, Burlington, ON, Canada). The resultant cDNA was diluted six-fold and kept at −20 °C for RT-PCR analysis.

The transcript levels of the target genes were analyzed using ABI StepOne Plus Real-Time PCR system (Applied Biosystems, Foster City, CA) and SYBR Green Real-Time PCR Master Mix (Toyobo, Japan) in 20 mL reactions. Each reaction mix contained 2 ng of total RNA, 0.5 μL of each primer (Supplementary Table S1) and 10 μL master mix. Reactions were carried out as follows: initial denaturation at 95 °C for 3 min, 38 cycles of 10 s at 94 °C, 20 s at 50–55 °C, and 20 s at 72 °C, followed by 5 min at 72 °C. For specific product verification, a melting curve was performed from 82 °C at 0.2 °C increments with a 10 s hold between observations. *YT521-B* gene was used as a standard control in the RT-PCR reactions. The relative expression of specific genes was quantitated with comparative Ct method as described earlier^54^. The experiments were repeated twice with three replicates.

### Histochemical staining for cytosolic calciumdetection, H_2_O_2_ and O_2_^−^

Labeling for cytosolic Ca^2+^ was performed by incubating cross-sections of the main root tissue with Fluo 3-AM (Molecular Probes, CAS#:121714-22-5) according to earlier reports^55^. Root tip was placed in 4 μM Fluo 3-AM dissolved in Hanks balanced salt solution (HBSS) containing 10 mM HEPES, 1mM Na_2_HPO_4_, 137 mM NaCl, 5 mM KCl, 1 mM CaCl_2_, 0.5 mM MgCl_2_, 5 mM glucose, 0.1% BSA, pH 7.4, and incubated at 37 °C for 60 min. And then the preincubated roots were washed with HBSS and fluorescence examination was performed using an IX70 inverted fluorescence microscope (Olympus, Japan), with the following settings: Ex 488 nm, Em 526 nm.

For histochemical detection of H_2_O_2_ and O_2_^−^, perennial ryegrass roots were stained with 3,3′-diaminobenzidine (DAB) and nitroblue tetrazolium (NBT), respectively, based on the method described by Zhang *et al.*^56^. The histochemical detection of H_2_O_2_ in perennial ryegrass roots was performed by directly immersion and infiltration of root quarters under vacuum with a freshly prepared DAB solution (1 mg mL^−1^, pH 3.8) at 25 °C in the dark for 30 min. For NBT staining to detect O_2_^−^ in roots, perennial ryegrass roots were immersed and infiltrated with 3.5 mg mL^−1^ NBT staining solution in 25 mM KOH-HEPES buffer (pH 7.8) and incubated in the dark for 30 min. After staining, roots were bleached in acetic acid-glycerol-ethanol (1/1/3, v/v/v) solution at 100 °C for 5 min, and then stored in glycerol-ethanol (1/4, v/v) until photographs were taken. H_2_O_2_ as a brown color and O_2_^−^ as a blue color were visualized.

### Metabolite extraction and derivatization

Metabolites from controls, Ca^2+^, H_2_O_2_, NaCl treated leaves and roots were extracted based on the procedure reported by Roessner *et al.*^57^ with some modifications. Frozen leaves and roots were ground to a fine powder in liquid nitrogen, transferred into 2 mL Eppendorf tubes, and then extracted in 1.4 mL of 80% (v/v) aqueous methanol under intensive oscillation (200 rmp) at 25 °C for 2 h. Then, a 50 μL methyl nonadecanoate (2 mg mL^−1^ in chloroform) with 20 μL internal standard (2 mg mL^−1^ ribitol in water) was added. Extraction was carried out at 70 °C in a metal bath for 15 min (each 5 min vortex 5 s). The tube was centrifuged for 5 min at 12 000 g. The supernatant was decanted to new 10-mL tubes, and 1.5 mL of water and 0.75 mL of chloroform were added. The mixture was vortexed thoroughly and subsequently centrifuged for 15 min at 8000 g. 300 μL polar phase (methanol/water) was decanted into 1.5 mL HPLC vials and dried in a benchtop centrifugal concentrator (Labconco Corporation, Kansas City, MI) overnight. The dried residue was redissolved and derivatized with 80 μL of 20 mg mL^−1^ methoxyamine hydrochloride in pyridine for 2 h under intensive oscillation (200 rmp) at 37 °C, and followed by a 2 h treatment at 37 °C with 50 μL N-methyl-N- trimethylsilyltrifluoroacetamide (MSTFA).

### Gas chromatography mass spectrometry (GC-MS) analysis

For GC-MS analysis, a 1 μL of the derivatization solutions was determined with a GC–MS (DSQII, Agilent 7890A/5975C, Hemel Hempstead, USA) system based on the method described by Hancock *et al.*^58^. Samples were injected into the DB5-MSTM column (15 m × 0.25 mm × 0.25 μm; J&W, Folsom, CA, USA) with a split ratio of 1:25. Injection temperature was set to 280 °C, the interface temperature was set at 290 °C, and the ion source temperature was adjusted to 200 °C. The column temperature was 5 min isothermal at 70 °C, then the GC oven temperature was raised to 260 °C with 5 °C min^−1^ in 2 min after injection, and finally held at 260 °C for 10 min. Helium gas was used as carrier set with a constant flow set at 1 mL min ^−1^. The MS measurement were set at electron impact (EI) source, electron energy 70 eV, solvent delay 4 min and the scan range 30–650 m/z at 0.6 scan s^−1^. Retention time (RT), retention indices (RI, http://gmd.mpimp-golm.mpg.de/search.aspx) and NIST Mass Spectral Database (version 11) were implemented to identify the target metabolites. Only metabolite detected at least three in five samples was considered true. Each metabolite was finally identified based on the internal consistency of RT and RI. For each sample in controls and Ca^2+^, H_2_O_2_, NaCl treated leaves and roots, we performed four biological replicate and two technical replicates per biological experiment.

### Statistical analysis

Statistical significance was performed by ANOVA using the Statistics Analysis System (SAS) (version 9.0 for Windows; SAS Institute, Cary, NC). Means for physiological measurements, gene expression and metabolites between treatments were separated using the Fisher’s least significant difference test (LSD) at *P* < 0.05 level.

## Additional Information

**How to cite this article**: Hu, T. *et al.* H_2_O_2_ and Ca^2+^-based signaling and associated ion accumulation, antioxidant systems and secondary metabolism orchestrate the response to NaCl stress in perennial ryegrass. *Sci. Rep.* **6**, 36396; doi: 10.1038/srep36396 (2016).

**Publisher’s note:** Springer Nature remains neutral with regard to jurisdictional claims in published maps and institutional affiliations.

## Supplementary Material

Supplementary Table S1

Supplementary Table S2

Supplementary Table S3

Supplementary Figure S1

Supplementary Figure S2
