# Supplementary material for: H2O2 and Ca2+-based signaling and associated ion accumulation, antioxidant systems and secondary metabolism orchestrate the response to NaCl stress in perennial ryegrass
Source: Sci Rep. 2016 Nov 2;6:36396. doi: 10.1038/srep36396 (PMC5090991; doi:10.1038/srep36396)
Supplement: Supplementary Table S1 [file srep36396-s1.doc]

**H_2_O_2_ and Ca^2+^-based signaling and associated ion accumulation, antioxidant systems and secondary metabolism orchestrate the response to NaCl stress in perennial ryegrass**

**Tao Hu**, **Ke Chen**, **Longxing Hu**, **Erick Amombo**, **Jinmin Fu**

**Table S1**

Primer sequences for RT-PCR ampliﬁcation analysis.

| Gene | Primers Sequences(5’-3’) | | Size (bp) |
| --- | --- | --- | --- |
| *Chl Cu/ZnSOD* | F | ATGGGTGCATATCDAYAG | 271 |
|  | R | GCCAGTCTTCCACCAGCAT |  |
| *Cyt Cu/ZnSOD* | F | GACACMACAAATGGHTGCAT | 221 |
|  | R | TCATCBGGATCGGCATGGACAAC |  |
| *MnSOD* | F | CAGRGBGCCATCAAGTTCAACG | 338 |
|  | R | TACTGCAGGTAGTACGCATG |  |
| *FeSOD* | F | TGCACTTGGTGATATTCCACTC | 297 |
|  | R | CGAATCTCAGCATCAGGTATCA |  |
| *CAT* | F | CCTSTCATTGTGMGTTTCTC | 292 |
|  | R | GTTAACTCCRAAVCCATCCATATG |  |
| *POD* | F | AGGCCCAGTGCTHCAMCTTC | 220 |
|  | R | TTGGTGTAGTAGGCGTTGTC |  |
| *pAPX* | F | CCTGAAAGGTCTGGGTTTGA | 173 |
|  | R | TCCTTGGCATAAAGGTCCAC |  |
| *GPX* | F | GCCGAGTATCCGATTTTTGA | 195 |
|  | R | TCGATACTGAGCGGAGAGGT |  |
| *GR* | F | TGTGCTGTTTTCTGCATTCC | 175 |
|  | R | AGTCTCAGCATCAACCACCA |  |
| *CaM1* | F | ATCACATCAGCACTCGCCAT | 186 |
|  | R | CGTTCATCTCCGACGACCTC |  |
| *CaM2* | F | TTCGCCACCAACTTCGTCCC | 169 |
|  | R | CACACATGCAGCAGCATCCA |  |
| *YT521-B* | F | TGT AGC TTG ATC GCA TAC CC | 112 |
|  | R | ACT CCC TGG TAG CCA CCT T |  |

*Chl Cu/ZnSOD*: chloroplastic *Cu/ZnSOD*; *Cyt Cu/ZnSOD*: cytosolic *Cu/ZnSOD*; F and R represent forward and reverse (the same below).
