# Supplementary material for: H2O2 and Ca2+-based signaling and associated ion accumulation, antioxidant systems and secondary metabolism orchestrate the response to NaCl stress in perennial ryegrass
Source: Sci Rep. 2016 Nov 2;6:36396. doi: 10.1038/srep36396 (PMC5090991; doi:10.1038/srep36396)
Supplement: Supplementary Figure S1 [file srep36396-s4.doc]

**H_2_O_2_ and Ca^2+^-based signaling and associated ion accumulation, antioxidant systems and secondary metabolism orchestrate the response to NaCl stress in perennial ryegrass**

**Tao Hu**, **Ke Chen**, **Longxing Hu**, **Erick Amombo**, **Jinmin Fu**


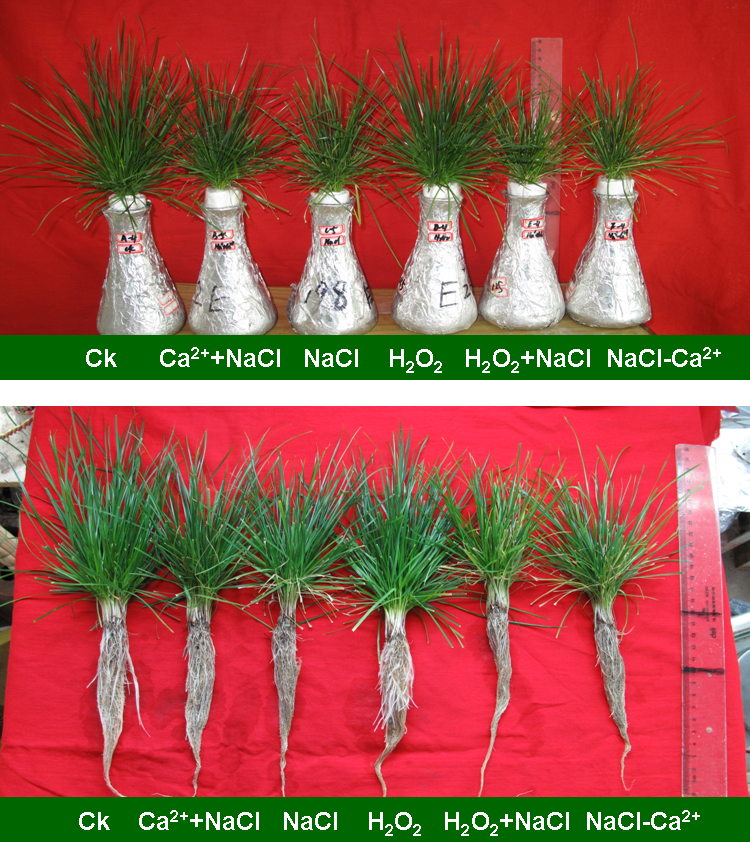


**Figure S1.**
