# Supplementary material for: H2O2 and Ca2+-based signaling and associated ion accumulation, antioxidant systems and secondary metabolism orchestrate the response to NaCl stress in perennial ryegrass
Source: Sci Rep. 2016 Nov 2;6:36396. doi: 10.1038/srep36396 (PMC5090991; doi:10.1038/srep36396)
Supplement: Supplementary Figure S2 [file srep36396-s5.doc]

**H_2_O_2_ and Ca^2+^-based signaling and associated ion accumulation, antioxidant systems and secondary metabolism orchestrate the response to NaCl stress in perennial ryegrass**

**Tao Hu**, **Ke Chen**, **Longxing Hu**, **Erick Amombo**, **Jinmin Fu**


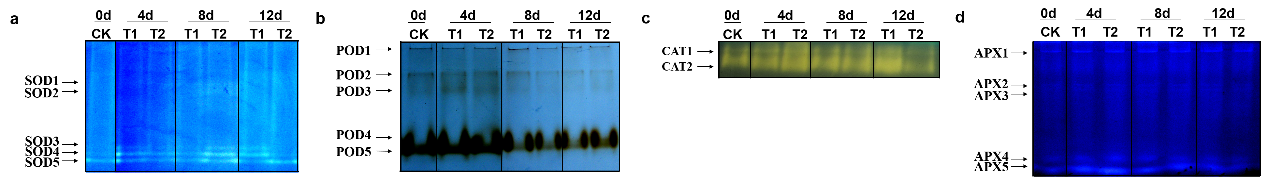


**Figure S2.** Exogenous Ca^2+^ modulates antioxidant-related isoenzymes in perennial ryegrass.

**The method**

Antioxidant isoenzymes were analyzed on native polyacrylamide gel electrophoresis (PAGE) with 5% of stacking gel and 10% of running gel with 50 μg protein per lane as described by Laemmli^1^. The displayed data is one of the representative results after all isoenzymes analyses were performed at least three times for each enzyme.

Staining for POD and SOD was carried out as described by Hu *et al*.^2^. The gel was incubated in 25 mM PBS (pH 7.0) for 15 min, then was immersed again in a freshly prepared 25 mM PBS (pH 7.0) containing 18 mM guaiacol and 25 mM H_2_O_2_ until POD activity-containing brown band visualized clearly. For SOD staining, the gels were incubated in 2.5 mM NBT dissolved in 50 mM PBS (pH 7.8) for 20 min in darkness at room temperature, then was washed three times with ice-cold distilled water and incubated in 36 mM PBS (pH 7.8) containing 28 μM riboflavin and 28 mM tetramethylethylenediamine (TEMED) for another 20 min. Finally, gels were immersed in 0.1 mM EDTA dissolved in 50 mM PBS (pH 7.8) and exposed to cool white fluorescent lamps (40 μmol m^−2^ s^−1^) at room temperature until the enzymes appeared as colorless bands in a purple background. Staining for CAT was performed according the method of Woodbury *et al*.^3^. The gels were incubated in 0.003% (v/v) H_2_O_2_ for 10 min and then stained in 1% potassium ferricyanide (w/v) and 1% ferric chloride solution (w/v) dissolved in distilled water until CAT activity-containing white band visualized clearly. Staining for APX was visualized by following the method described by Hu *et al*.^17^. The gels were submerged in 50 mM PBS (pH 7.0) containing 2 mM ascorbate for 30 min, followed by a 20-min incubationin in a solution composed of 50 mM PBS (pH 7.0), 4 mM ascorbate and 2 mM H_2_O_2_ for 30 min. Finally, the gels were washed with 50 mM PBS (pH 7.8) containing 28 mM TEMED and 2.45 mM NBT for 10–20 min with gentle agitation in the presence of light until the enzymes appeared as an achromatic band on a purple-blue background.

**The result**

Salinity-induced profile changes of antioxidant enzymes were observed by Native- PAGE-electrophoresis. Five SOD (SOD1, SOD2, SOD3, SOD4, SOD5), five POD (POD1, POD2, POD3, POD4, POD5), two CAT (CAT1, CAT1), and five APX (APX1, APX2, APX3, APX4, APX5) isoforms were detected (Figure 3a–d, inset plates). These partly reflected the observed changes in enzymatic activities above (Figure 3a–d). Salinity increased the intensity of the three isoenzymes of SOD (3–5), whereas greater intensity was found to occur under Ca^2+^ application at 4 and 12 DAT. However, at 8 DAT, SOD3, SOD4 and SOD5 intensity was depressed in Ca^2+^ treated plants. In addition, SOD1 and SOD2 isoenzymes were determined in 8 days NaCl-treated plants. The intensity of the four isoenzymes of POD (2–5) decreased with the increasing NaCl-stressed time, while higher intensity of the four isoenzymes was observed in salt-treated leaves under Ca^2+^ feeding at 8 DAT compared with NaCl alone-treated ones. No differences in POD1 isoenzyme intensity were observed among all the treated plants. During the first four days treatment, no differences in two CAT (CAT1, CAT2) isoenzymes were found. However, the addition of Ca^2+^ to NaCl-treated plants increased CAT1 and CAT2 intensity at 8 and 12 DAT. In addition, application of Ca^2+^ upon salinity imposition enhanced APX2 and APX3 intensity at 4 DAT but decreased at 8 DAT in comparison with NaCl treatment. When plants were exposed to salt stress for 12 days, all APX (APX1, APX2, APX3, APX4, APX5) isoenzymes had greater intensity for the application of Ca^2+^.

1. Laemmli, U. K. Cleavage of structural proteins during the assembly of the head of bacteriophage T4. *Nature* **227,** 680-685 (1970).
2. Hu, L., Li, H., Pang, H. & Fu, J. Responses of antioxidant gene, protein and enzymes to salinity stress in two genotypes of perennial ryegrass (*Lolium perenne*) differing in salt tolerance. *J. Plant Physiol*. 169, 146-156 (2012).
3. Woodbury, W., Spencer, A. & Stahmann, M. An improved procedure using ferricyanide for detecting catalase isozymes. *Anal. Biochem*. **44,** 301-305 (1971).
